# Essential Genes to Consider in Epstein-Barr Virus-Associated Gastric Cancer: A Systematic Review

**DOI:** 10.7759/cureus.11610

**Published:** 2020-11-21

**Authors:** Ana S Armenta-Quiroga, Raheela Khalid, Paramvijay Singh Dhalla, Jian Garcia, Anusha Bapatla, Arunima Kaul, Safeera Khan

**Affiliations:** 1 Internal Medicine, California Institute of Behavioral Neurosciences & Psychology, Fairfield, USA; 2 Medicine, California Institute of Behavioral Neurosciences & Psychology, Fairfield, USA

**Keywords:** stomach neoplasms, epstein-barr virus, viral genes

## Abstract

Gastric cancer (GC) is a prevalent malignancy worldwide; the Epstein-Barr Virus (EBV) also affects many people worldwide. An important association has been seen in these two diseases that could explain causality and a possible viral etiology of GC as has been seen with *Helicobacter pylori*. This study aims to identify genes expressed in malignant cells that are infected with EBV and see if one could be more oncogenic than the other. We conducted a systematic review based on the preferred reporting items for systematic reviews and meta-analysis (PRISMA) guidelines. We had 29 observational studies after inclusion/exclusion criteria and quality assessment for every single study. A total of 1022 patients were evaluated for different types of genes in 29 papers. It was demonstrated that the most expressed genes or the gene most involved were genes that are seen in Epstein-Barr virus-associated gastric cancer (EBVaGC) as latent genes of the EBV-infected cells, which are found in tumor cells. The genes that were mostly involved were LMP2, BNLF2a, and the absence of LMP1 that lead to the expression of BARF1, among other genes. These studies were made on mostly Asian populations, so it is still unknown if these genes involved have a geographical association more than an EBV and GC association.

## Introduction and background

Epstein-Barr virus-associated gastric cancer (EBVaGC) is 10% of all gastric carcinomas, representing the fifth-most common cancer worldwide and the third-most deadly cancer [1, 2].

Gastric cancer is a multifactorial disease that is associated with several risk factors that could contribute to the cause of this disease. This cancer has been linked to the presence of *Helicobacter pylori*, which suggests an infectious etiology related to this malignancy [1]. There are variations in incidence between countries, age, and sex. That is why it is also believed that because it's a multifactorial disease, the genetic part plays an essential role in developing gastric cancer [2, 3].

On the other hand, the Epstein-Barr virus is characterized by its double-stranded DNA and belonging to the herpes virus family [4]. It is a virus that has infected approximately 90% of adult individuals and is known for being self-limiting. In a few cases, it has been distinguished by developing malignancy of lymphoid and epithelial origin [5, 6]. This virus can remain latent in the B lymphocytes of the infected individual [4].

EBVaGC has important molecular characteristics because of the expression of different genes and its epigenetic profile [7]. It is believed that the Epstein-Barr virus could be another infectious agent that contributes to the transformation of gastric cells into malignant cells due to different cellular processes and signaling pathways [8].

As mentioned, an infectious etiology has been suggested. The mechanism of *Helicobacter pylori *and gastric cancer is very well known; however, another infectious agent has been identified as significantly associated with this type of cancer. The specific variables of the Epstein-Barr virus that cause this cancer and the expressed genes that are involved are still not well understood, and it is still unknown if any of these is more oncogenic than the others.

Today, the different genes associated with gastric cancer are still being investigated. Likewise, the mechanisms and pathways that produce overexpression or loss are studied [9, 10]. There is already a bit of information about new therapeutic approaches considering the evidence on the subject [11].

This study aims to search for the specific genes of the Epstein-Barr virus that are expressed in gastric cancer and determine if any of these is more oncogenic than the other. Studying this is very useful since these genes can eventually serve as a target for the specific treatment of the disease.

## Review

Methods

Study Design

We conducted a systematic review based on the preferred reporting items for systematic reviews and meta-analysis (PRISMA) guidelines.

Eligibility Criteria

We selected all observational studies related to this topic, Epstein-Barr virus, gene expression, and gastric cancer, for the last 10 years. We included all types of studies found in full text and done in humans, excluding all those studies in animals. The articles included were in the English and Spanish languages. Finally, gray literature and narrative review were excluded.

Databases and Search Methods

We used the PubMed database to search for relevant studies for our data collection. This search was done between the 9th and the 13th of July 2020. For this, we used the medical subject headings (MeSH) Keyword. First, we searched "Stomach Neoplasm," "Herpes 4, Human" and "Epstein-Barr Virus Nuclear Antigens," which showed 110,438, 13,075, and 3,377 results, respectively. We then decided to use a MeSH Strategy instead of a more effective search of articles related to the topic. The combined keywords we used in this strategy were the following: "Stomach Neoplasms/microbiology"[MeSH] AND "Herpesvirus 4, Human"[MeSH] AND "Viral Matrix Proteins"[MeSH], "Stomach Neoplasms/microbiology"[MeSH] AND "Epstein-Barr Virus Nuclear Antigens"[MeSH] and "Stomach Neoplasms/microbiology"[MeSH] AND "Herpesvirus 4, Human/pathogenicity"[MeSH] AND "Proteins"[MeSH].

Study Selection

The eligibility process is shown in Table 1, which represents the evolution of the use of inclusion/exclusion criteria and screening process to select studies.

**Table 1:** MeSH strategy used in the literature search Inclusion/exclusion criteria and screening process for article eligibility.

MeSH Strategy	Database	Before Inclusion/exclusion criteria (results)	After Inclusion/exclusion criteria (results)	After the screening process
"Stomach Neoplasms/microbiology"[Mesh] AND "Herpesvirus 4, Human"[Mesh] AND "Viral Matrix Proteins"[MeSH]	PubMed	52	12	11
"Stomach Neoplasms/microbiology"[Mesh] AND "Epstein-Barr Virus Nuclear Antigens"[MeSH]	PubMed	42	14	10
"Stomach Neoplasms/microbiology"[Mesh] AND "Herpesvirus 4, Human/pathogenicity"[MeSH] AND "Proteins"[MeSH]	PubMed	40	13	8

After the first search, we had 134 articles, and once we applied the inclusion-exclusion criteria, we had 40. We excluded all the articles that were duplicated and not relevant to the topic of the screening process. We excluded four articles because they were not pertinent and seven because of duplicates in the search giving us 29 papers.

Risk of Bias and Quality Assessment

To reduce the risk of bias, we did a quality assessment of every paper to avoid this risk. We used the New-Castle-Ottawa questionnaire to assess all of the observational studies.

Results

**Figure 1:**
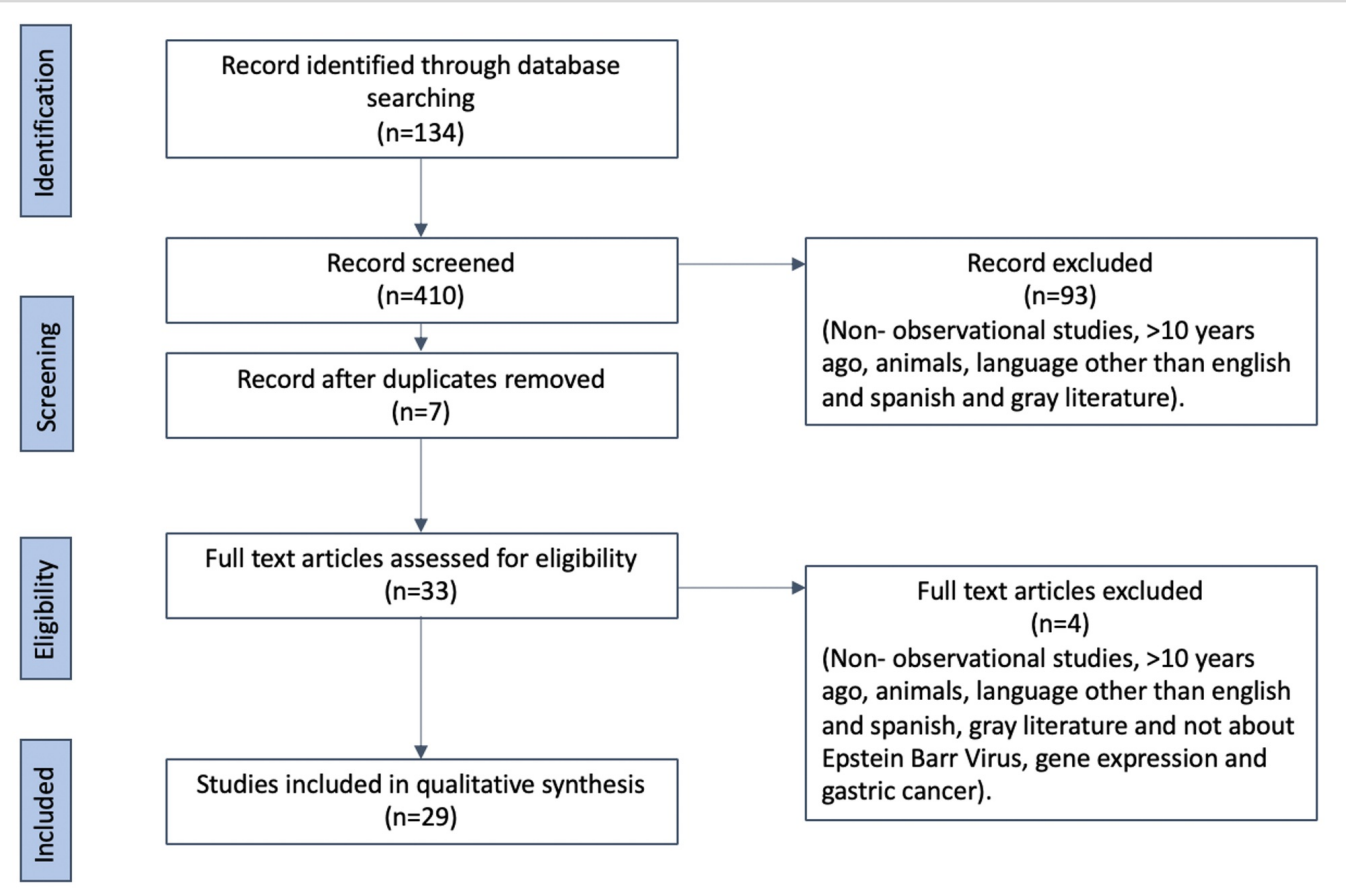
PRISMA flow diagram.

The search results focused on observational studies that discussed specific genes that were expressed in EBVaGC. This was done to point out genes present in Epstein-Barr virus and see if any is more oncogenic than the others, especially for the evolution of gastric cancer. After we used the inclusion/exclusion criteria, 29 articles were obtained from which the data were abstracted. Figure 1 shows the evolution of the search.

From the 29 observational studies, 15 cohort studies and 14 cases and controls were obtained. A quality assessment was carried out of these studies, getting favorable results. Of the 29 papers, we evaluated 1022 patients for the expression of a different gene. As shown in Table 2, all the included studies are mentioned, the type of study of each one, year of publication, country, and the genes studied by paper. These articles include samples from countries such as China, Japan, Korea, Thailand, United States, India, Portugal, Iran, Canada, and Zambia. It is worth highlighting the fact that most of the population studied in these articles is Asian.

**Table 2:** Qualitative aspects of the included studies

	Author	Year of publication	Type of study	Country	Gene
1	Armero et al. [7]	2017	Cohort	USA	EBNA1
2	Chang et al. [12]	2013	Case Control	Korea	BARF1
3	Chen et al. [13]	2012	Case Control	China	EBER1, EBNA1
4	Cho et al. [14]	2018	Cohort	Japan	PD-L1
5	Han et al. [15]	2012	Cohort	China	LMP2, EBER1
6	He et al. [16]	2015	Case Control	China	EBER1, p16, FHIT, CRBP1, WWOX, DLC-1
7	Rymbai et al. [3]	2015	Case Control	India	EBNA1
8	Kayamba et al. [17]	2016	Case Control	Zambia	EBNA1
9	Kosari-monfared et al. [18]	2019	Cohort	Iran	CTNNBIP-1
10	Luo et al. [19]	2012	Case Control	China	EBER1, EBNA3-C, gp350/220
11	Moon et al. [20]	2017	Case Control	Korea	PD-L1
12	Nakayama et al. [21]	2019	Cohort	Japan	EBNA1, PD-L1
13	Ribeiro et al. [22]	2017	Cohort	Portugal	LMP2, EBER
14	Liu et al. [23]	2015	Cohort	China	EBER1, BNLF2a, BNLF2a-A, BNLF2a-B1
15	Shinozaki-Ushiku et al. [24]	2015	Cohort	Japan	BART4-5p
16	Sivachandran et al. [25]	2012	Case Control	Canada	EBER, EBNA1
17	Strong et al. [26]	2015	Cohort	USA	BNLF2a
18	Sundar et al. [27]	2018	Case Control	Korea	EBER, PD-L1
19	Wang et al. [28]	2016	Case Control	China	LMP1, EBER1, VEGF-C, BARF1
20	Wang et al. [10]	2019	Cohort	China	LMP2, DNMT3a
21	Wang et al. [29]	2010	Cohort	China	LMP2, EBER1
22	Wang et al. [30]	2013	Case Control	China	EBER1, EBNA3-A
23	Kawazoe et al. [31]	2017	Case Control	China	EBER1, EBNA2-A, EBNA2-B, EBNA2-C
24	Wanvimonsuk et al. [32]	2019	Case Control	Thailand	LMP1, EBER
25	Wu et al. [33]	2012	Cohort	China	EBER1, EBNA3-C
26	Liu et al. [34]	2016	Cohort	China	LMP2, EBNA1, BZLF1
27	Zhang et al. [35]	2015	Case Control	China	LMP2, EBER1, HER2
28	Zhao et al. [36]	2013	Cohort	China	LMP1, EBER, DNMT1, DNMT3b
29	Zhao et al. [2]	2016	Cohort	China	GPC4

**Figure 2:**
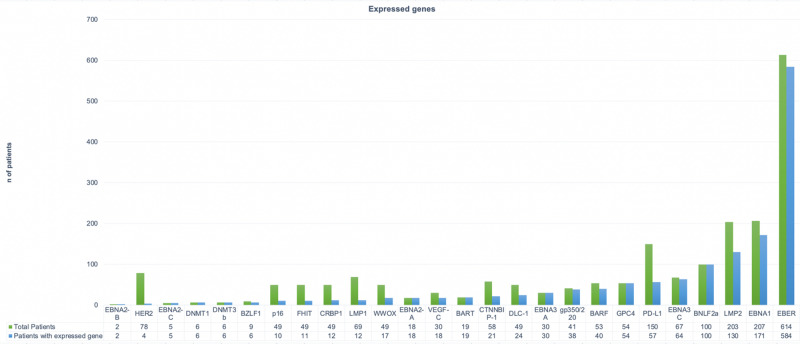
Comparison of the number of patients evaluated by each gene and the total number of patients who expressed the said gene.

At first glance the article summary demonstrates that the genes that are most seen in EBVaGC are the latent genes of the EBV-infected cells, which are found in tumor cells. In Figure 2, we see the comparison made between the number of evaluated patients per gene and the number of patients who express that gene. The most expressed genes are shown on the right side.

Discussion

Genes Expressed in Latently Infected Cells

Most of the EBVaGC patients studied were sought for association with genes expressed in latently infected cells; infected cells which showed a higher detection of EBER, EBNA 1, EBNA3A, and LMP2. As shown in Figure 2, most patients express the Epstein-Barr virus encoded-small RNAs (EBERs), however this only means that the virus is present in the malignant cells since it represents the non-coding RNA associated with Epstein-Barr virus. It is essential to recognize this fact because it means that the presence of the virus in gastric cells can be an important factor in the development of malignancy [27].

An additional possible oncogenic expression may include the latent membrane protein 2 (LMP2) expression that has been seen to induce hypermethylation of various factors through up-regulation that has enormous carcinogenic potential. Ribeiro et al. state that LMP2 causes up-regulation of DNMT1 and phosphorylation of STAT3 [22]. Wang et al. mention the hypermethylation of AQP3, and Zhao et al. talk about the up-regulation of DNMT3b, which causes hypermethylation of almost 886 genes that are involved in cancer-related pathways [10, 36]. It is essential to recognize that the presence of LMP2 leads to many different pathways with the potential to develop a malignancy. The fact that it is present in so many patients suggests that it is an essential factor that could cause EBVaGC.

Studies by Wang et al. and Han et al. have shown that PY and ITAMs regions are highly conserved in LMP2, suggesting that they play an essential role in the viral infection. These regions are considered crucial for possible treatment options targeted LMP2 directed explicitly to these regions. Epitope mutations still have to be considered, this will make target immunotherapy more challenging, but it is an excellent place to start [15,30].

Regarding LMP1, it has been determined that it has an oncogenic activity. According to Wanvimonsuk et al., it has been noted that it is expressed in a decreased way or absent in this disease [28,32]. However, according to Wang et al., the presence of LMP1 leads to the expression of BARF1, which might also play a role in the development of malignant cells [28].

Finally, the Epstein-Barr virus nuclear antigen (EBNA) expression shows that it is more common in most patients to express EBNA1 and EBNA3C. Moreover, EBNA1 has been seen to have an essential role in malignancy development. Armero et al. mention that it interacts with cellular splicing factors, and Nakayama et al. say that it induces the expression of PD-L1, which plays a vital role in diseases such as cancer [7,21]. This suggests that it is a possible factor that can contribute as a cause of EBVaGC [21]. However, it was seen by Chen et al. that polymorphisms can have geographic-associated polymorphisms rather than tumor-specific mutations, which is why it is essential to do more studies in different patients to determine if this is true [13]. Likewise, EBNA3C is known to be a geographic-associated polymorphism in China [33].

Other Expressed Genes

Other expressed genes that were seen with greater frequency were BNLF2a, PD-L1, GPC4, and BARF. BNL2a is a gene expressed in EBV with the function of viral evasion against HLA I, giving T-cell immunity. In some studies, it has a protective role in latently infected tumor cells [26]. It was seen that type A, BNLFaA, is dominant in a study done in the Chinese population, and B1, BNLF2aB1, is geographically restricted to this population. BNLFa expression in the patients seen in Liu et al. study demonstrated a highly conserved gene that gives us another option for targeted immunotherapy. However, it is essential to consider that more studies should be made to see if it is highly conserved in patients from other countries [23].

The presence of PD-L1 has been detected in patients' tumor cells in some studies; however, it's common to see the expression of this gene in many different types of cancer. It is not a specific gene for EBVaGC, but the overexpression of PD-L1 does regulate some signaling pathways with malignant potential [14,21]. Even though it's not specific for this malignancy, it is an immunotherapy option for many cancers [14]. A recent paper by Sundar et al. studies LMP1 expression. Even though LMP1 expression is higher in EBVaGC, it states that EBVaGC with lower expression of PD-L1 in tumor cells leads to the worst prognosis, which is also an excellent point to consider [27].

There is not much information about GPC4 expression. Still, it was seen in Zhao et al. study that in a Chinese population, there is a polymorphism of this gene that may represent a risk factor given by the susceptibility associated with this polymorphism to develop EBVaGC [2]. Nevertheless, we come back to the same issue; it is unknown if this polymorphism is of the Chinese population only and if the susceptibility changes depending on the population.

Finally, BARF1 is another crucial factor to consider. This is because in the absence of LMP1, as it was already mentioned, BARF1 is expressed, causing its protein to promote the proliferation of EBV-infected malignant gastric cells. It does this through some pathways that may have essential functions in gastric cancer tumorigenesis. In the papers that studied the expression of this gene, in most cases, the expression of BARF1 was found. Then again, more studies are needed to determine this factor as a possible cause [12,28].

The rest of the genes mentioned before may play a role in malignancy development; however, this role is not found in most cases. This means that even though they are essential, they are not the priority since it could not help a general population, only specific patients. Perhaps when more is known about EBVaGC and the most common genes involved, it will be useful to research these genes.

This study had limitations mostly since it is very little published evidence on the subject. This could lead to publication bias; however, the data abstraction and review were done from a complete database available at the time. Another limitation is that most of the patients studied are from the Asian population; however, there could be variations between populations that we do not see or dominant polymorphisms in the different populations that predominate in the Asia population and differ from those of the rest. The lack of information only prompts more studies to be carried out in other populations.

## Conclusions

Different genes were studied and identified in patients with EBVaGC. We can conclude that latent EBV genes were mostly expressed in malignant cells such as LMP2, BNLF2a, and the absence of LMP1 showed that lead to the expression of BARF1, among other genes. It is important to emphasize that most of the patients are Asian, so it is unknown if these genes could be associated with this disease in this population only. It would be recommended in the future to carry out more studies in patients from different countries to determine whether these genes are expressed in EBVaGC of all patients or only of this population.
